# Medical Literature, Etc.

**Published:** 1878-04-20

**Authors:** 


### *EDITORIAL AND MISCELLANEOUS.*

☞All communications relating to the business of The Record, for the years 1877 and 1878, must be addressed to DR. R. C. WORD, Managing Editor Southern Medical Record, Atlanta, Ga.

☞Brief and practical communications are solicited on all subjects pertaining to medicine; also reports of cases in practice.

☞Send money by check, postal order or registered letter.

☞Write your name, post-office, county and State plainly.

### MEDICAL LITERATURE—STATE MEDICAL ASSOCIATIONS, ETC.

In a calm review of the progress of medical literature in the last decade, there is much to gratify the devotee of science and the lover of truth. At no period of the world's history has there been a greater number of profound students and practical investigating minds than at the present time. Germany, it is claimed, is at the van of progress, especially in respect to physiological researches. France is also active in the field of investigation; and in our own land we are scarcely behind either of the countries named in the pursuit and development of any department of scientific truth. In the department of therapeutics especially, it is conceded, we believe, that the United States is in advance of the world.

Touching the progress of medical literature, we claim that in the United States we are second to no other country. Journalism is at a high standard, and is constantly developing.

Medical Associations are numerous and active. The transactions that emanate from these organizations in several of the States are of a high order, exhibiting the presence of many learned and investigating minds. In some of the Southern States we have to regret that too little attention, comparatively, has been given to medical literature. While we claim as great talent and as much practical knowledge in proportion to numbers as exists in the Northern section of the Union, it is nevertheless true that the journals of our section, though mostly of a high order, and very practical, are not sustained as they should be by the Southern profession. Our State societies, too, with rare exceptions, are in a great degree neglected. In certain of the States unfortunate divisions have existed and still exist. The absence of harmony is ever attended with a want of interest. Without united action and fraternal feeling, little or nothing can be accomplished. Controversies at times may be expected, and divisions, if upon principle, are right and often unavoidable. If error obtrudes itself it must be opposed; controversies must be met and adjusted. But they must be met in the right way. If those who lead and direct in these associations are guided by just, honorable and unselfish principles, it is easy to settle difficulties. But when a favored few take the entire control, ignoring the deserving but modest members of the profession, or when *assessments are made which bear too heavily upon the brethren whose pecuniary circumstances are limited,* then the association will not and can not flourish. If divisions exist, they should be harmonized by fair, impartial and honorable action. Adjustments, to be satisfactory, must be full and complete.

Cordial co-operation can not be expected of those who, though being acknowledged right, see the principles they advocate ignored or discarded. Nor is a charge of obstinacy just against those who insist on a strict adherence to principle. Adjustments which compromise with error and embrace the advocates of wrong, may be accepted by men of policy, but carry with them no true or permanent harmony, and meet with no response in the hearts of those who are guided alone by sincere and honest opposition to error.

If, then, unfortunate divisions exist, and, as in some places they do; if distrust has taken root and discord is perpetuated, the responsibility rests upon those who have sought peace at the sacrifice of principle.

